# Multimodal assessment of circadian sleep health in predicting mental health outcomes in adolescents

**DOI:** 10.3389/frsle.2023.1177878

**Published:** 2023-11-28

**Authors:** Kara McRae Duraccio, Sarah Kamhout, Isabella D. Wright, Kathleen Erekson Rugh, Jack Miskin, McKenna Amdal

**Affiliations:** Department of Psychology, Brigham Young University, Provo, UT, United States

**Keywords:** circadian alignment, DLMO, actigraphy, chronotype, mental health, depression, adolescence

## Abstract

**Introduction:**

Aspects of circadian sleep health including circadian alignment, circadian phase, or chronotype may be related to mental health outcomes in adolescents. Using novel and robust data collection methods, this study explored the relationship between adolescents' circadian sleep health and traits related to depression, anxiety, stress, and emotional regulation.

**Methods:**

Fifty-two healthy 14–18-year-olds (58% female; 94% European American) participated in this study. Across a 10-day period, participants completed wrist-worn actigraphy. Next, participants completed a dim-light melatonin onset (DLMO) protocol where 12 saliva samples were collected over a 6-h period to measure circadian phase. Circadian phase was calculated as the duration of time between DMLO to average sleep onset time across the monitoring period. Social jetlag was measured as the discrepancy between sleep times from weekday to weekend. Participants completed the Depression Anxiety Stress Scales (DASS-21), Emotion Regulation Questionnaire (ERQ), and the Morningness-Eveningness Questionnaire for Adolescents (MEQ). Following dichotomizing sleep outcomes into clinically relevant groups (late vs. early circadian phase, aligned vs. misaligned circadian rhythms, minimal social jetlag vs. presence of social jetlag, intermediate to morningness vs. eveningness chronotype), we conducted general linear models to determine circadian group differences in mental health outcomes (depression, anxiety, stress, expressive suppression, and cognitive reappraisal) while controlling for gender and pubertal development.

**Results:**

Circadian phase had a large effect on depression symptoms in adolescents, with adolescents with later DLMO having significantly higher depression scores than those with earlier DLMO (*p* = 0.031). Chronotype had a medium but non-significant effect on anxiety and stress symptoms in adolescents, with adolescents with eveningness-tendencies having higher anxiety and stress symptoms than those with intermediate to morningness-tendencies (*p*'s = 0.140 and 0.111, respectively).

**Conclusions:**

In the first ever study using gold-standard methodologies to examine the relationship between mental health and circadian sleep health in healthy adolescents, we observed that adolescents with later circadian phase had increased depressive symptoms compared to earlier circadian phase. Furthermore, adolescents who endorsed behaviors that suggest eveningness tendencies may have heightened stress/anxiety. These conclusions encourage future experimental research regarding this topic and may help inform interventions aimed to decrease depression, anxiety, and stress in adolescents.

## Introduction

Global estimates suggest that 10–20% of children and adolescents will experience a mental health condition before adulthood [Evaluation I of HM and Global Health Data Exchange (GHDx), 2021]. The United States' Center for Disease Control and Prevention found a 33% increase in youth endorsing feelings of persistent hopelessness between 2008 and 2021 (Centers for Disease Control and Prevention, 2023). Such symptoms can have profound effects on adolescents' daily functioning and quality of life (Jaycox et al., 2009; Geng et al., 2020; Petito et al., 2020). Tragically, in 2021, 22% of adolescents reported seriously considering suicide (Centers for Disease Control and Prevention, 2023). Between 2008 and 2015, hospital visits for active suicidality nearly doubled, with adolescent girls being most strongly represented in this shift (Plemmons et al., 2018). Fortunately, previous studies have found that early interventions are associated with improved symptom prognosis not only within the adolescent period, but also across the lifespan (Das et al., 2016; McGorry and Mei, 2018; Petito et al., 2020). Therefore, understanding the factors that can increase or decrease rates of mental illness is vital to youth's current and future wellbeing.

Given its strong association with psychological and physical health across previous studies, sleep may be an especially salient yet clinically and culturally overlooked influencing factor of adolescent mental wellness (Campbell et al., 2012; Tarokh et al., 2016; Galván, 2020; Kuhlman et al., 2020; Lucien et al., 2021). Previous research suggests that sleep may be an early indicator of mental health difficulties, as well as serve as an accessible treatment target for individual therapies and public health initiatives (Blake et al., 2018; Petito et al., 2020). Sleep may help prevent some symptoms of mental distress due to its proposed impact on emotional regulation and the HPA axis (Palmer and Alfano, 2017; Blake et al., 2018; Nicolaides et al., 2020). Despite strong evidence suggesting that adequate sleep is also protective factor against depressive, anxious, and suicidal symptoms (Pasch et al., 2010; Lovato and Gradisar, 2014; Zhai et al., 2015; Ojio et al., 2016; Robillard et al., 2018), it is estimated that <30% of adolescents are receiving the 8–10 h of sleep of sleep recommended for their age group (Paruthi et al., 2016; Wheaton, 2018), with most adolescents only sleeping an average of 7.4 h per night (Galland et al., 2018) and more than 40% of US adolescents sleeping <7 h per night (Twenge et al., 2017). Disturbances in an adolescent's sleep pattern can impact the development of psychiatric conditions during this period as well as later in life (Tesler et al., 2013). To complicate matters further, these increases in mental health disorders have the potential to negatively impact sleep (Gregory and Sadeh, 2012; Baron and Reid, 2014; Lovato and Gradisar, 2014). This suggests that earlier improvements in sleep may lead to greater improvements in mental health outcomes over time.

Adolescents are at the highest risk of obtaining short and ill-timed sleep compared to any other developmental group (Crowley et al., 2018). This increased susceptibility can be due to social pressures placed on school-aged adolescents, early school start times, extracurricular activities being scheduled in the early morning hours (selected by administrators within older age groups with different sleep needs) (Dunster et al., 2018), and biological changes in intrinsic circadian rhythms and homeostatic sleep processes (Crowley et al., 2015; Logan and McClung, 2019). Some adolescents may also use sleep to exert their autonomy, delaying sleep to spend more with friends, catch up on homework, or browse social media (Power et al., 2017). Together, these factors can lead to reduced sleep duration.

Short sleep has long been associated with poorer mental health. While the majority of research demonstrating the negative impact of short sleep on mental health has been cross-sectional in nature (Short et al., 2019), a large two-wave study found that short sleep predicted anxiety disorders at 1 year follow-up, but baseline anxiety did not predict short sleep (Roberts and Duong, 2017), suggesting that short sleep may be a modifiable causal factor of anxious symptoms, rather than an unavoidable side effect of their presence. Beyond anxiety, a review of 73 studies in adolescents also found that reduced sleep was associated with a 55% increase in likelihood of mood difficulties (Short et al., 2020). Indeed, pooled data from over 500,000 adolescents demonstrates a curvilinear relationship between short sleep and suicidality, with 8–9 h of sleep per night being associated with the lowest risks of suicidal ideation or attempts in this age group (Chiu et al., 2018).

In addition to impacting sleep duration, adolescents' social demands can also clash with their sleep chronotype, or the preference a person has for morningness (sleeping early and waking early) or eveningness (sleeping late and waking late) behavioral patterns. Preference for eveningness typically peaks in adolescence and declines as individuals reach young adulthood, with males typically experiencing a later peak than females (Fischer et al., 2017; Randler et al., 2017a). Due to its association with daily behaviors, chronotype is most frequently measured via self-report measures (Levandovski et al., 2013). Self-reported eveningness has been associated with increased symptoms of depression across the lifespan (Hasler et al., 2010; Kivelä et al., 2018; Bauducco et al., 2020) as well as increased prevalence of seasonal affective disorder in adults (Tonetti et al., 2012). In adolescents, eveningness has been linked with lower self-regulation (Owens et al., 2016) and broad depressive symptoms (Chan et al., 2020; Chen et al., 2021) but less consistently with anxiety or suicidality (Alvaro et al., 2014; Díaz-Morales, 2016; Haraden et al., 2019; Chan et al., 2020; Chen et al., 2021; Kuula et al., 2022), despite previous studies identifying such associations in adults (Gaspar-Barba et al., 2009; Rumble et al., 2020; Bradford et al., 2021; Mokros et al., 2021). There is debate as to why mood may be negatively impacted by evening chronotype. It is thought that eveningness may be more associated with negative mental health outcomes due to pleiotropic impacts of genes associated with both sleep disorders and changes in neuronal development (Veatch et al., 2017). It is also possible that individuals with more evening chronotypes are at greater risk of short sleep due to circadian misalignment, or the mismatch between circadian phase and current sleep patterns. As societal demands tend to favor earlier chronotypes, adolescents with later chronotypes may be especially susceptible to effects of circadian misalignment on mental health, on top of the sleep debt risks already inherent to their age group.

Physiological measures of circadian timing can also provide insights into endogenous patterns of sleep and wakefulness, known as circadian phase. The suprachiasmatic nucleus (SCN) of the brain mediates the entrainment of internal circadian patterns with external light cues. Dim Light Melatonin Onset (DLMO) protocols, which aim to identify the onset of melatonin secretion, are considered the gold standard for assessing human circadian phase (Lewy, 1999). Existing studies in youth have used DLMO to identify associations between later circadian phase and higher negative and lower positive affect in a general sample (Dolsen and Harvey, 2018) as well as shorter melatonin secretion periods and worsened mood in adolescents with high BMIs (Simon et al., 2020). These findings suggest that adolescent preference for eveningness, as well as later endogenous circadian phases, increase risk for mood disorders, potentially due to circadian misalignment and sleep debt that are inherent in this developmental group.

Indeed, circadian misalignment may be the driving force behind the relationship between later chronotype and circadian phase and mental health. Several studies have demonstrated the relationship of circadian misalignment with mental health outcomes (Pasch et al., 2010; Winsler et al., 2015; Bauducco et al., 2020), though none (to our knowledge) have utilized gold-standard methods of assessing circadian misalignment to address this gap in the literature in community samples of this age group. In adolescents with diagnosed mood disorders, greater DLMO-assessed circadian misalignment was associated with greater depressive symptoms (Robillard et al., 2018). Circadian misalignment has also been linked with increased depressive symptoms in adults with major depressive disorder (Emens et al., 2009; Coleman et al., 2019), increased atypical symptoms of depression such as changes in appetite in pre- and peri-menopausal women (Meliska et al., 2011), and depression ratings in adults with Seasonal Affective Disorder (Lewy et al., 2006). These findings suggest that similar patterns may also be evident in general population samples of adolescents with subclinical symptom profiles.

One specific type of circadian misalignment that has received a deal of attention in its relationship to mental health in youth is “social jetlag” - the discrepancy between sleep timing on work/school days vs. free days. In the context of the adolescent population, social jetlag generally presents as students sleeping very little on weekdays due to later circadian phase and early school start times and then attempting to catch up on missed sleep on the weekends via falling asleep late and sleeping late into the morning (when school does not necessitate early wake times) (Baron and Reid, 2014). Social jetlag has historically been measured using self-report methods where the difference of midsleep between work/school days and “free days” are calculated (Jankowski, 2017). Using this calculation, greater self-reported social jetlag has been linked with increased depressive and anxious symptoms in adolescents (Mathew et al., 2019); however, social jetlag has yet to be robustly assessed alongside physiological measures of circadian misalignment in the context of anxiety and mood-related symptoms (Henderson et al., 2019).

Taken together, these findings suggest the potential associations between circadian sleep health (involving chronotype, circadian phase, and circadian alignment) and mental health in adolescents, though more studies examining these relationships simultaneously are needed. Methodologies are important to consider since subjective report of sleep behaviors has been linked to increased bias (Biddle et al., 2015; Cespedes et al., 2016) and ±1.5 h differences in circadian phase estimates (Lovato et al., 2016). However, physiological methods of assessing circadian phase can be costly and time intensive. If more affordable means of measuring circadian sleep health are sufficient to detect risk of mental health difficulties in this age group, they may be well suited to more widespread clinical use in diagnosis and treatment monitoring, even outside of research settings. However, if widely utilized circadian-related surveys are not as sensitive to increased risk of mental health difficulties as physiological measures (i.e., DLMO), this may suggest a need for improved self-report measures or increased referral and insurance support for formal DLMO procedures.

In response to the need for greater understanding of sleep as a risk factor for negative mental health outcomes in adolescence and to identify affordable and accessible methodologies to assess circadian alignment in this cohort, we used multi-modal methods of calculating circadian sleep health (i.e., DLMO, actigraphy, and self-report) to investigate how various aspects of adolescent sleep correspond with mental health symptoms. We compared relationships between physiological measures of circadian phase to those of self-report measures of chronotype and actigraphy-assessed social jetlag (weekday/weekend-specific changes in sleep timing). We hypothesized that increased self-reported levels of depression, stress, and anxiety, as well as decreased self-reported levels of emotional regulation ability, would be predicted by:

1. Later circadian phase (assessed through DLMO)
2. Evening chronotype (assessed through the morning-eveningness questionnaire; MEQ)
3. Greater levels of social jetlag (assessed through actigraphy)
4. More extreme circadian misalignment (assessed as whether the time elapsed between DLMO and average sleep onset falls outside of the expected range)

Findings from this study may inform future assessments for mental health disorders in this age group and provide insight into aspects of circadian sleep health which are most relevant to treatment of particular mental health symptom clusters.

## Materials and methods

This analysis was conducted as part of a larger sleep study using actigraphy, DLMO, and MRI data to explore the effects of adolescent circadian misalignment on executive function and obesity risk. All procedures in this study were approved by the Brigham Young University Institutional Review Board (IRB2021–306).

### Participants

Healthy adolescents ages 14–18 years old were contacted through lunchroom recruitment booths at high schools near the sponsoring organization, printed flyers posted on university and high school bulletin boards, and digital flyers in schools' email newsletters. Interested adolescents completed an online survey to determine eligibility based on age and current enrollment in school. Due to an MRI protocol employed during other phases of this project, adolescents with metal implants, significant claustrophobia, or history of traumatic brain injury were excluded. As this study was also part of a larger examination of obesity-related factors, individuals whose BMIs were in the obese range (BMI%iles > 95%ile) as well as those who took medication known to interfere with diet or sleep behaviors (i.e., antidepressants, sleep medications) were also excluded.

### Procedures

All baseline appointments were held on a Tuesday or a Wednesday. During baseline appointments, parental consent and participant assent were obtained and participants were given a sleep diary to track their time spent in bed as well as their perceived sleep and wake times over a 10-day monitoring period. Participants were also taught how to wear and care for a wrist-worn accelerometer (ActiWatch 2) for nighttime use (time entering bed with intention to sleep until leaving the bed in the morning). Participants then completed a measure about their preferred sleeping timing, known as sleep habits including the Morningness-Eveningness Questionnaire (MEQ). The PDS (Pubertal Development Scale) was also administered during this phase to account for differences in circadian alignment associated with biological maturation.

For the first 7 days of the monitoring period, adolescents were instructed to sleep as they normally would; for nights 8–10, adolescents were asked to self-select a “typical” bedtime and waketime (a time that they felt their body would naturally want to fall asleep and wake up at) that they would adhere to across the three nights. This method was selected to stabilize sleep in a developmental population that faces highly variable sleep (Crowley et al., 2018) to enhance the likelihood of obtaining a valid DLMO estimate. Between the baseline and the assessment appointments, participants received daily text reminders to wear the accelerometers and to complete the sleep diary. Participants were also reminded of their assessment appointment time and of the sleep/wake times for nights 8–10 that were determined during the baseline appointment.

Following the 10-day monitoring period, participants attended the assessment appointment, where they returned their completed sleep diary and accelerometer. After completing an MRI scan for a different segment of the project, they spent 6 h in a dimly lit sleep lab for DLMO procedures where they were required to provide a saliva sample every 30 min. Throughout the 6-h session, participants filled out measures of emotional wellbeing, including the Emotion Regulation Questionnaire (ERQ) and the Depression, Anxiety, and Stress Scale – 21 Items (DASS-21). Due to the low-light environment, these measures were administered via paper and pencil and in large-print font. Between completing surveys and providing saliva samples, participants were free to participate in pleasant, low-light activities, including playing board games or solving puzzles, or playing video games or watching shows/movies on a dimly lit television. Applicable questions within measures were used to screen for current suicidality; any indicative responses were addressed with a suicide assessment conducted by a trained clinician or supervised graduate student. At the conclusion of the DLMO appointment, participants were compensated according to their adherence to and participation in study procedures.

### Measures

#### Covariates

##### Demographics

To account for covariates that may relate to affect or sleep behaviors, age, sex, race, ethnicity, height, weight, medication use, date of last menstrual period, and familial income were voluntarily collected via an online survey at the baseline appointment.

##### Pubertal Development Scale (PDS)

We assessed for level of pubertal maturation using the Pubertal Development Scale (PDS) (Robertson et al., 1992). Given that most adolescents undergo a shift toward later circadian alignment (evening chronotype) following puberty, with chronotype generally not returning to prepubertal levels until youths' mid-to late- twenties (Crowley et al., 2018), it is important for studies of adolescent sleep to assess whether each subject has undergone puberty and is therefore subject to greater endogenous circadian misalignment. The PDS has been shown to be a reliable and valid measure of physical development (ICC = 81–0.92, Cronbach's Alpha = 0.91–0.96) (Koopman-Verhoeff et al., 2020). PDS is thus comparable measure to physical assessments performed by healthcare professionals such as Tanner staging, but its self-report format is more comfortable for participants and more conducive to research settings (Koopman-Verhoeff et al., 2020). Scores were included as a covariate. Internal consistency for this sample was adequate at 0.70.

#### Primary outcomes

##### Emotional regulation questionnaire (ERQ) for children and adolescents

We used the Emotional Regulation questionnaire (ERQ) for Children and Adolescents to conceptualize our primary outcome of emotional regulation (Gross and John, 2003; Gullone and Taffe, 2012). This 10-item survey looks at two facets of emotional regulation: (1) cognitive reappraisal, or how an individual's definition of a stressor can change its emotional impact, and (2) expressive suppression, or how much someone inhibits emotionally expressive behaviors over time. In a general community sample of over 1,000 Australian adults, the ERQ has been found to have strong internal consistency reliability (Cronbach's Alpha = 0.89–0.90 for cognitive appraisal, = 0.76–0.80 for expressive suppression) (Preece et al., 2020). The revised version for children and adolescents also displayed strong internal consistency (Cronbach's Alpha = 0.82–0.86) and moderate 12-month stability (ICC = 0.37–0.47 for cognitive reappraisal, = 0.40–0.63 for expressive suppression) (Gullone and Taffe, 2012). Within our sample, our Cronbach's Alpha for the cognitive reappraisal facet was good at 0.82 and the expressive suppression facet was fair at 0.60.

##### Depression, anxiety, and stress scale – 21 items (DASS-21)

Depressive, anxious, and stress-associated primary outcomes were measured using an abbreviated version of the Depression Anxiety and Stress Scale (DASS-21) (Lovibond and Lovibond, 1995). These subscales have been found to be reliable in adolescent populations with Cronbach's Alpha measuring 0.87 for depression, 0.79 for anxiety, and 0.83 for tension/stress (Szabó, 2010); within our sample, our Cronbach's Alpha was 0.83.

#### Measures of circadian dimensions

##### Chronotype

Chronotype was measured with the Morningness-Eveningness Questionnaire (Horne and Östberg, 1976). This 19-item, multiple choice, self-report survey was used to determine participant chronotype, which we analyzed as a dichotomous predictor of mental health outcomes. The MEQ asks subjects to identify when they most prefer to wake up and go to bed, as well as their peak times of functioning. Total scores range from 16 to 86, with scores below 41 suggesting an evening chronotype and scores above 59 suggesting a morning chronotype. Although validation studies of the MEQ have not specifically looked at the adolescent population, several studies that include young adults showed strong internal consistency [Cronbach's α = 0.81–0.86 (Thun et al., 2012; Inomata et al., 2014; Treven Pišljar et al., 2019); within our study sample, the Cronbach's alpha was adequate at 0.70]. To facilitate interpretation, we dichotomized the sample into two groups: eveningness-tendencies (MEQ total score ≤ 41) and intermediate to morningness-tendencies (MEQ total score ≥42).

##### Social jetlag

Social Jetlag was measured using sleep estimates gathered by actigraphy. Actigraphy was used with the knowledge that adolescents are notoriously poor at estimating their sleep habits (Bauer and Blunden, 2008). Further, there is good evidence of actigraphy serving as a strong estimate of social jetlag in pervious literature (Malone et al., 2016; Crowley et al., 2018; Cespedes Feliciano et al., 2019; Lucas-Thompson et al., 2021). Specifically, a wrist-worn accelerometer (ActiWatch 2) was initialized to collect data in 30 s epochs. Participants were instructed to wear the accelerometer at night, beginning when they entered bed with the intention of falling asleep. Participants also completed sleep diaries, where they self-reported the times that they entered their bed with the intention to sleep, fell asleep, woke up, and stepped out of bed. Information recorded in sleep diaries was used to verify sleep and wake onset times and to guide scoring in the actigraphy data. Data from wrist-worn actigraphs has been shown to correlate with polysomnography (*r* =0.81–0.83); however, specificity for wake times is weaker (Meltzer et al., 2012; Quante et al., 2018). The Sadeh algorithm (Sadeh et al., 1994) was used to calculate sleep onset, sleep offset, and sleep midpoint, and sleep duration.

We used an updated social jetlag calculation that accounts for the degree of weekend oversleep to produce a social jetlag estimate independent of sleep debt. Specifically, we used the following equation to calculate social jetlag: SJLsc = |sleep onset on free days – sleep onset on weekend days| (Jankowski, 2017). Using this estimate of SJL, we then dichotomized the sample into two groups: minimal social jetlag (≤1 hour) or presence of social jet lag (>1 h). This cutoff was selected as a shift in as little as 1 h in sleep patterns (as in the case of daylight savings time) can have a negative impact on youth health (Johnson and Malow, 2023).

##### Circadian misalignment

Circadian phase was assessed through salivary Dim Light Melatonin Onset (DLMO) (Benloucif et al., 2008) during a six-hour assessment appointment that occurred on a Friday or Saturday evening. This assessment was scheduled to begin 5 h before and end 1 h beyond their predetermined typical bedtime that was selected at the baseline appointment. We used this self-selected typical bedtime to inform the assessment window as previous research has demonstrated that lack of personalization of this assessment window can lead to high missingness of DLMO (Keijzer et al., 2011). The assessment took place in a dimly-lit room (<5 lux at eye-level, confirmed every 30 min via hand-held luxometer) where DLMO salivary samples were collected. Specifically, each 30 min, participants were instructed to soak a cotton swab with saliva for ~2 min, or until the cotton swab was saturated, for a total of 12 saliva samples across the 6 h period. After each collection, the sample was weighed; samples that did not meet processing requirements (<2 mL) were disposed of and participants immediately provided a replacement sample. Samples >2mL were time-stamped, centrifuged at 3000 g for 5 min to remove the salvia from the cotton swab, and frozen for analysis. Frozen samples were then shipped to and processed by SolidPhase Inc., an external lab that uses Novolytix RIA kits to measure salivary melatonin concentrations. In order to minimize potential contamination, during the 10 min prior to each sample collection, adolescents refrained from eating or drinking and ensured that their mouths were clean by brushing their teeth with a soft bristle toothbrush while relaxing in a reclined position.

Our lab used a threshold of 4 pg/mL to determine saliva melatonin concentration values, a threshold that has been previously validated in adolescent populations (Carskadon et al., 1997). Adolescents were categorized into two circadian phase groups: early timing (DLMO prior to 10:00 pm) or late timing (DLMO at 10:00 pm or later). This cut point was determined based on previously established mean DLMO times in later adolescents (Crowley et al., 2014) and because having a DLMO time of 10:00 pm or later would suggest a physiologically appropriate bedtime of midnight or later (based on mean phase angle reported in later adolescents) (Crowley et al., 2014) that may increase the likelihood of sleep restriction due to early school start times. Circadian alignment (also referred to as phase angle) was calculated as the difference in time between DLMO and the average sleep onset time that was recorded via actigraphy over the 10-day monitoring period. Following determine the phase angle of adolescents, we categorized the sample into two groups: “aligned,” representing cases where there was a phase angle of ≥ 2 h between DLMO and the average sleep onset time (reflecting the anticipated delay between DLMO and sleep initiation in low-light conditions) (Pandi-Perumal et al., 2007; Crowley et al., 2014) and “misaligned,” signifying instances where the a time gap of <2 h (indicating adolescents initiating sleep onset before their physiological readiness). No adolescent in our study had a sleep onset phase angle gap of >4 h, which would have also been considered “misaligned” (indicating adolescents falling asleep later than their body's natural readiness).

### Data preparation

#### Actigraphy

ActiWatch software (Actiware 6.3) was used to download wrist-worn accelerometer data in 30 s poch intervals. Sleep period onset and offset data was cross verified between actigraphy measurements and sleep diaries. In the event that sleep onset or waketime was not noted in a participant's sleep diary (*N* = 2) or, in cases of noticeable discrepancies between the sleep diary and the ActiWatch log (i.e., significant physical activity after diary suggested they had fallen asleep or significant inactivity before the diary suggested they had fallen asleep (*N* = 2), two reviewers jointly estimated in-bed and out-of-bed times. In these 4 instances, in-bed time was defined as the beginning of the last downward trend of physical activity prior to sleep, and out-of-bed time was defined as the beginning of the first prolonged period of activity following sleep-associated inactivity. We used the ActiWatch software to clean the data and the Sadeh et al. (1994) algorithm was used to generate a report of sleep onset and offset and sleep duration. Midsleep was calculated as the halfway point between sleep offset and sleep onset.

### Statistical analyses

We conducted an *a priori* power analysis using G^*^Power 3.1.9.7 and found that to conduct a linear multiple regression (random model) to test three sleep predictors (i.e., chronotype, social jetlag, and circadian alignment) as well as three covariates (to be determined at time of analyses), we needed a sample size of 48 to have 0.80 power to detect a large effect with a 0.05 alpha error probability. Under the assumption that we would have at least four instances of equipment failure (i.e., accelerometers not recording sleep, DLMO sample loss), we aimed to recruit 52 adolescents.

Data were analyzed using SPSS (Version 28, IBM Inc., Chicago, IL). First, sample characteristics were generated using both means (SD) and frequencies. Meaningful covariates included in our models included sex assigned at birth (given frequently observed sex differences in anxiety and mood-related mental illnesses) (Yoon et al., 2023), pubertal status (given that transition through puberty is often associated with increased mental illness) (Pfeifer and Allen, 2021), and income (as this may be a factor related to social determinants of sleep health in adolescents) (Hawkins and Takeuchi, 2016). We then conducted a series of general linear models that tested whether mental health outcomes (anxiety, stress, depression, or emotional regulation facets) differed across four aspects of circadian health, including chronotype group (morningness vs. eveningness), timing (early vs. late DLMO), circadian alignment (aligned vs. misaligned), and social jetlag (minimal social jet lack, presence of social jet lag). We determined that the assumptions for these models were met prior to analyses. To determine statistical significance, a two-tailed *p*-value of 0.05 was selected. We utilized partial eta squared ($\eta_{p}^{2}$) as an estimate of effect size; specifically, partial eta squared informs how large of an effect the independent variable has on the dependent variable (with of 0.01, 0.06, and 0.14 indicating small, medium, and large effect, respectively). Due to our smaller sample size, we interpreted research findings if the estimated $\eta_{p}^{2}$ was medium (0.06) or larger.

## Results

### Participants

A total of 52 adolescents completed the experimental procedures [mean age 15.54, SD = 1.37; 58% female (sex assigned at birth); 94% European-American; see Table 1 for detailed demographic characteristics]. Of these 52 adolescent participants, 43 had valid accelerometry data for estimation of circadian misalignment and social jetlag (there were 5 instances of accelerometer non-compliance and 4 instances of equipment malfunction). Additionally, 47 participants had valid DLMO estimates for estimating circadian misalignment (3 instances where saliva melatonin concentrations did not exceed 4 pg/ml and 2 instances where there no instances where concentrations fell below 4 pg/ml). All 52 participants were able to complete the required measures.

**Table 1:** Participant demographics.

**Demographics**	**Mean ±SD or %**
N	52
Age	15.54 ± 1.37
Pubertal Development Score (PDS^*^)	12.69 ± 4.18
Female (sex assigned at birth) (%)	58
**Race/Ethnicity (%)**	
European-American	94.0
Other	6.0
**Income (%)**	
$20,000-$49,000	1.6
$50,000–$74,000	16.4
$75,000–$99,000	14.8
$100,000–$149,000	27.9
$150,000 or more	24.6
Declined to report	14.8
**Mental health characteristics**	
Depression (DASS-21^**^)	11.13 ± 5.88
Anxiety (DASS-21^**^)	9.86 ± 7.76
Stress (DASS-21^**^)	13.70 ± 5.80
Emotional regulation – cognitive reappraisal	18.90 ± 5.02
Emotional regulation – expressive suppression	15.67 ± 3.71
**Sleep characteristics**	
Circadian alignment (phase angle)	1:45 ± 1:14
Dim light melatonin onset	21:30 ± 1:11
Morningness/eveningness preference (MEQ^***^)	47.15 ± 6.48
Social jet lag	0:40 ± 1:19

* Pubertal Development Scale.

** Depression, Anxiety, and Stress Scale, 21-item version.

*** Morning-Eveningness Questionnaire.

As summarized in Table 1, participants presented with (on average) mild levels of depression (mean = 11.15, SD = 5.88) and anxiety (mean = 9.86, SD = 7.76) and normal levels of stress (mean = 13.70, SD = 5.80). Based on established DASS-21 cut-offs, 13 participants had moderate levels of depression, 2 participants had severe levels of depression, and 1 had extremely severe levels of depression. Furthermore, 10 participants had moderate anxiety, 1 had severe anxiety, and 9 had extremely severe anxiety. Finally, 10 participants had moderate levels of stress, and 1 had severe levels of stress. Normal distributions were observed in circadian alignment (phase angle; ranging from 0:09 to 3:52), DLMO (ranging from 19:00 to 24:30), MEQ total score (ranging from 35 to 68), and social jetlag (0:05–3:34).

### Circadian phase

**Figure 1:**
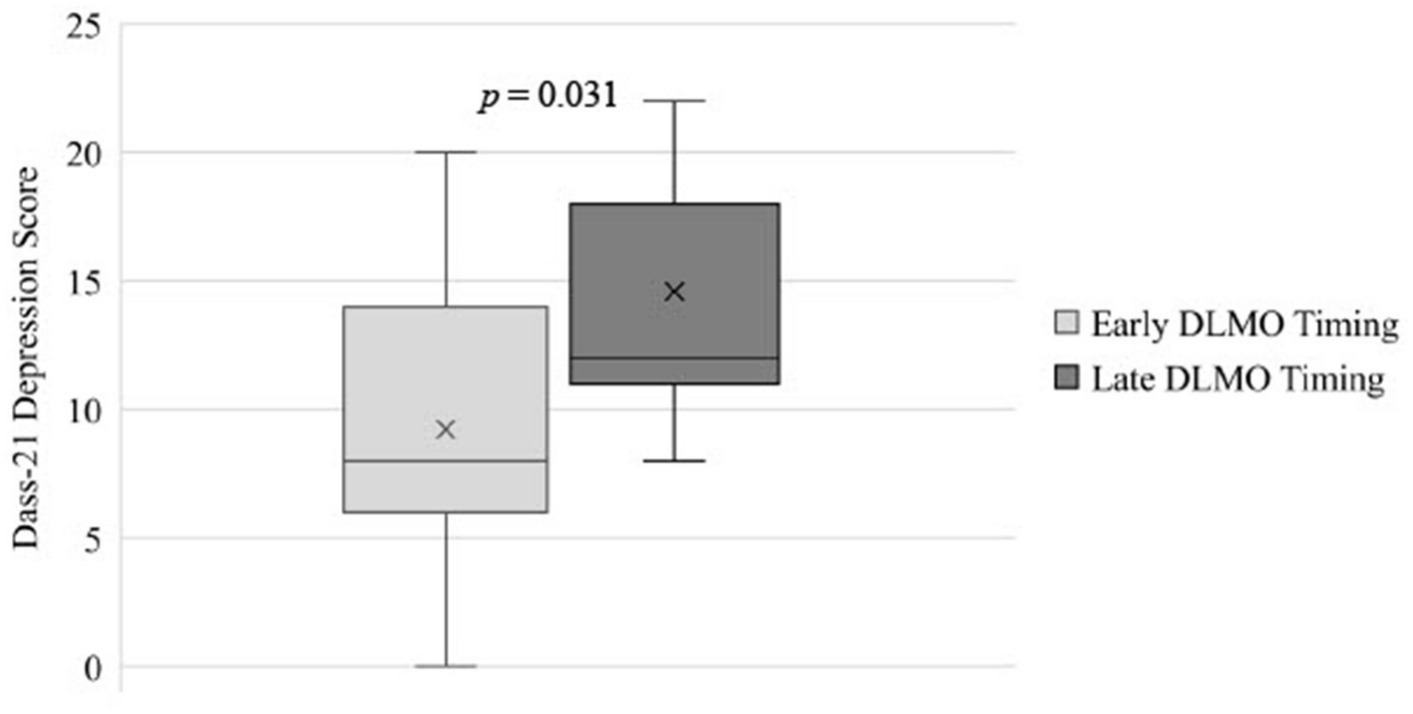
Differences in depression scores for adolescents with earlier DLMO vs. Later DLNIO timing. DLMO, Dim Light Melatonin Onset; DASS-21, The Depression, Anxiety, and Stress Scale — 21 Items.

Circadian phase had a large effect on depression symptoms in adolescents, with adolescents with later DLMO having significantly higher depression scores (M = 14.89, SD = 7.62) than those with earlier DLMO (M = 9.24, SD = 5.14; *p* = 0.031; $\eta_{p}^{2}$ = 0.13; see Table 2 and Figure 1). Differences in anxiety, stress, or emotional regulation facets across circadian phase groups were small ($\eta_{p}^{2}$ ≤ 0.02) and non-significant (*p*'s > 0.432).

**Table 2:** Group differences in obesity-related outcomes amongst early circadian phase vs. late circadian phase.

	**Early DLMO (*N =* 29)**		**Late DLMO (*N =* 9)**		**F**	***p***	$\eta_{p}^{2}$
	**M**	**SD**	**M**	**SD**			
**Circadian phase**							
Depression	9.24	5.14	14.89	7.62	5.07	0.031	0.13
Anxiety	8.97	8.29	10.89	6.94	0.06	0.802	0.00
Stress	13.17	6.38	14.00	5.48	0.00	0.994	0.00
Cognitive reappraisal	19.55	5.10	18.11	2.85	0.63	0.432	0.02
Expressive suppression	15.24	3.81	15.56	3.71	0.06	0.812	0.00
	**Morningness tendency (*****N** =* **30)**		**Eveningness tendency (*****N** =* **11)**		**F**	***p***	$\eta_{p}^{2}$
	**M**	**SD**	**M**	**SD**			
**Chronotype**							
Depression	9.93	5.13	12.91	7.76	0.99	0.326	0.03
Anxiety	8.60	7.43	13.45	8.49	2.27	0.140	0.06
Stress	12.60	6.22	16.55	4.39	2.67	0.111	0.07
Cognitive reappraisal	18.87	4.51	18.55	6.12	0.02	0.897	0.00
Expressive suppression	15.40	3.68	15.18	3.60	0.01	0.912	0.00
	**Minimal SJL (*****N** =* **20)**		**Presence of SJL (*****N** =* **18)**		**F**	***p***	$\eta_{p}^{2}$
	**M**	**SD**	**M**	**SD**			
**Social jet lag (SJL)**							
Depression	10.60	4.64	10.67	7.55	0.23	0.637	0.01
Anxiety	10.20	7.25	9.22	8.06	0.00	0.974	0.00
Stress	13.80	5.46	12.89	6.37	0.05	0.830	0.00
Cognitive reappraisal	18.50	4.37	19.0	5.56	0.08	0.780	0.00
Expressive suppression	15.65	3.76	15.06	3.64	0.30	0.588	0.01
	**Aligned (*****N** =* **15)**		**Misaligned (*****N** =* **20)**		**F**	***p***	$\eta_{p}^{2}$
	**M**	**SD**	**M**	**SD**			
**Circadian alignment**							
Depression	9.07	5.12	11.50	7.04	0.78	0.385	0.03
Anxiety	8.13	8.12	10.00	7.17	0.52	0.477	0.02
Stress	12.40	5.41	13.50	6.42	0.25	0.622	0.01
Cognitive reappraisal	18.67	5.68	19.60	3.82	0.57	0.458	0.02
Expressive suppression	14.93	3.81	15.65	3.87	0.24	0.627	0.01

### Chronotype

**Figure 2:**
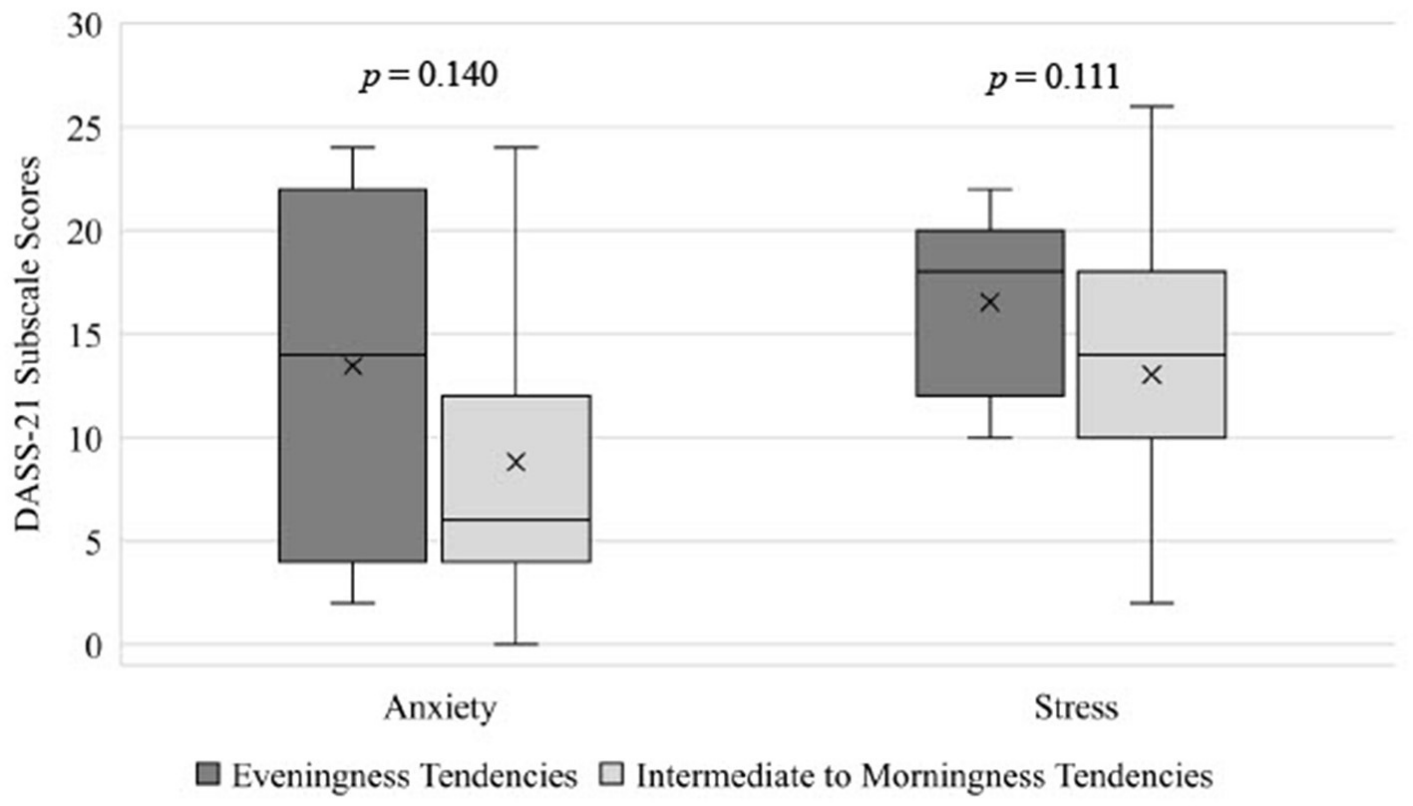
Differences in anxiety and stress in adolescents mith eveningness tendencies and intermediate to morningness tendencies, estimated from the MEQ. DASS-21, The Depression, Anxiety, and Stress Scale — 21 Items; MEQ, Momingness/Eveningness Questionnaire.

Chronotype had a medium but non-significant effect on anxiety symptoms in adolescents, with adolescents with eveningness-tendencies having higher anxiety scores (M = 13.45, SD = 8.49) than those with intermediate to morningness-tendencies (M = 8.60, SD = 7.43; *p* = 0.140; $\eta_{p}^{2}$ = 0.059; see Table 2 and Figure 2). Additionally, adolescents with eveningness-tendencies trended toward having higher levels of stress (M = 16.55, SD = 4.39) than those with intermediate to morningness-tendencies (M = 12.60; SD = 6.22; *p* = 0.111, $\eta_{p}^{2}$ = 0.069; see Table 2 and Figure 2). Differences in depression or emotional regulation facets across chronotype groups were small ($\eta_{p}^{2}$ ≤ 0.027) and non-significant (*p*'s > 0.326).

### Social jetlag

No differences in depression, anxiety, stress, or emotional regulation facets across SJL groups were noted ($\eta_{p}^{2}$ ≤ 0.009; *p*'s > 0.588).

### Circadian misalignment

No differences in depression, anxiety, stress, or emotional regulation facets across circadian alignment groups were noted ($\eta_{p}^{2}$ ≤ 0.025; *p*'s > 0.385).

## Discussion

Given the imperative role sleep plays in the development and maintenance of mental health pathology in adolescents, research examining more granular areas of sleep may be vital to early intervention, prevention, and effective treatment (Blake et al., 2018; Petito et al., 2020). This study aimed to examine the relationship between aspects of sleep circadian health and mental health symptomology in adolescents. While considering carefully considering unique nature of circadian phase, chronotype, and circadian alignment, we employed multi-modal and gold-standard physiological measurement of circadian phase (actigraphy and DLMO) along with self-report measures of chronotype and psychological wellbeing to thoroughly investigate potential connections between circadian health and mental health.

We observed that even after accounting for sex and pubertal status, adolescents with later circadian phase (as measured by DMLO) had increased depressive symptoms. This corroborates previous research findings that indicate that later circadian phase is associated with higher negative affect in youth (Alvaro et al., 2014). Similarly, recent research has examined the effectiveness of a behavioral intervention aimed to advance bedtime earlier into the evening (the Transdiagnostic Sleep and Circadian Intervention; TranS-C) on sleep and depression outcomes in adolescents. Specifically, one pilot trial in adolescents with ADHD (Becker et al., 2022) and a randomized control trial in adolescents with comorbid evening circadian preference and depression (Asarnow et al., 2023) demonstrated that TranS-C improved adolescent depression symptomatology following the intervention, and improved circadian alignment may be the mechanism that reduces depressive symptoms (Asarnow et al., 2023). These findings, paired with adult recommendations on how to control light levels across the 24 h day to help align circadian rhythms (Brown et al., 2022), provide compelling evidence that incorporating interventions that aim to advance sleep timing and promote circadian alignment in adolescents may serve as a powerful target for reducing depression.

Alternatively, our combined models did not replicate previous associations between adolescent circadian misalignment (Robillard et al., 2018), social jetlag (Mathew et al., 2019), or chronotype (Tonetti et al., 2012; Owens et al., 2016; Chan et al., 2020) and mental health outcomes; however, our findings neared significance for chronotype on anxiety and stress, where adolescents with identified eveningness-tendencies had greater symptoms of both anxiety and stress, compared to adolescents with identified morningness-tendencies. We hesitate to interpret non-significant findings but acknowledge that despite lacking statistical significance (which may be due to our smaller sample size), the differences in anxiety and stress had a medium effect size. Should our research findings be replicated using larger sample sizes (Watts and Norbury, 2017), these findings would corroborate fMRI research that demonstrates that later chronotype is associated with reduced connectivity between the amygdala and the cingulate cortex (Horne and Norbury, 2018), a circuit previously associated with high neuroticism (Cremers et al., 2010). This may suggest a neural basis for associations between genetic or temperamental sleep preferences and emotional responsiveness. However, given the mixed cross-sectional and longitudinal findings on chronotype and anxiety in adolescents (Power et al., 2017; Haraden et al., 2019), future research on this topic is warranted.

It is worth noting that genetic traits related to circadian rhythm development and maintenance also influence neural development or connectivity involved in mental health (Veatch et al., 2017). Such individual genetic variance may explain why some adolescents may be especially susceptible to negative effects of short or misaligned sleep. The hypothesis that different trends may emerge for different genetic or behavioral circadian profiles would be in alignment with previous findings on adults with delayed sleep phase disorder, which point to significant heterogeneity in impacts of circadian factors on sleep and mental health symptoms (Murray et al., 2017). This may suggest that circadian phase and misalignment may be more impactful on mental health in some groups than others, even when focusing on those who already demonstrate significant clinical sleep concerns.

Another significant area of heterogeneity and individual variance in associations between sleep and mental health is personality. Many traits such as extraversion, openness, agreeableness and conscientiousness have been associated with morning chronotype (Randler et al., 2017b). Of particular relevance to this context, neuroticism has also been associated with evening chronotype (Randler et al., 2017b). This is in alignment with our findings that adolescents with later chronotype trended toward having higher levels of anxiety and stress. Eveningness, lower levels of conscientiousness, and higher levels of neuroticism have also been associated with greater symptoms of depression (Gorgol et al., 2022). Future studies could examine personality as a moderating factor in this age group.

To our knowledge, this is the first study to use DLMO to examine circadian phase and circadian misalignment in conjunction with mental health outcomes in a population of adolescents from the general community. A key strength of this study is its naturalistic observation of sleep behaviors during the weekend minimal manipulation of participants' typical bedtime routines (excluding the three-night stabilization period prior to DLMO assessment). Additionally, our multimodal assessment of circadian health allows for a comprehensive view of sleep timings' potential influence on affect. Our focus on physiological measures may explain some differences between our results and those of past investigations focusing on self-report.

A primary limitation of this study is our small sample size, which reduced power of all analyses. This small sample size was chosen based on MRI outcomes being included in the primary analyses of the larger project wherein this data was collected. Future investigations would benefit from increased sample sizes which may facilitate increased sensitivity. This may be especially important to consider in regard to our lack of observed effects in the combined models as well as our lack of observed relationships between any circadian-related measurement and adolescents' stress or anxiety levels. Due to our focus on multiple methods of measurement as well as multiple mental health symptom clusters, we conducted multiple analyses, which increases our likelihood of Type I error. Our study design was also cross-sectional, meaning that even correlations identified as statistically significant should not be used to infer causality. The same can be said for studies using DLMO to study circadian alignment at large, although relevant longitudinal studies have been published (Haraden et al., 2019; Chen et al., 2023). Future studies could also use manipulation protocols to further confidence in suggested trends.

Additionally, actigraphy measurement only spanned a seven-day period, increasing the likelihood of altered SJL estimates; future studies should consider implementing a longer collection period. Further, in our attempt to stabilize sleep to increase the likelihood of obtaining a valid DLMO sample, we may have inadvertently altered the naturalistic sleep patterns of adolescents and the DLMO estimate; future research should consider broadening the DLMO assessment window to allow adolescents the flexibility of sleeping naturalistically leading up to the appointment while still maximizing odds of obtaining valid DLMO samples. Finally, while our participants were recruited from the general community, there was limited ethnic, geographic, and socioeconomic variability in our sample that limits the generalizability of our findings. Further, the sample included healthy adolescents which likely limited the severity of mental health difficulties within our sample which may have limited our ability to detect effects that may be present in more clinical cohorts. Future studies should investigate whether these findings generalize to more representative adolescent populations.

## Data availability statement

The raw data supporting the conclusions of this article will be made available by the authors, without undue reservation.

## Ethics statement

The studies involving humans were approved by the Brigham Young University Institutional Review Board (IRB2021–306). The studies were conducted in accordance with the local legislation and institutional requirements. Written informed consent for participation in this study was provided by the participants' legal guardians/next of kin.

## Author contributions

KD served as the principle investigator on this project, oversaw data collection, analyzed statistics, and contributed to manuscript preparation. SK helped prepare the data and significantly contributed to manuscript preparation. IW participated in data collection, helped prepare the data, analyzed statistics, and contributed to writing the manuscript. KR helped with data collection and contributed to the manuscript. JM and MA with preparing the data and contributed in writing the manuscript. All authors contributed to the article and approved the submitted version.
